# Lgr5 expression is a valuable prognostic factor for colorectal cancer: evidence from a meta-analysis

**DOI:** 10.1186/s12885-015-1986-2

**Published:** 2016-01-12

**Authors:** Yangyan Jiang, Wenlu Li, Xin He, Hongbo Zhang, Fangzhen Jiang, Zhigang Chen

**Affiliations:** Department of UItrasonic diagnosis, Second Affiliated Hospital, Zhejiang University School of Medicine, Hangzhou, China; Department of Pharmacology, Zhejiang University School of pharmacy, Hangzhou, China; Department of Hematology, Second Affiliated Hospital, Zhejiang University School of Medicine, Hangzhou, China; Department of Neurology, Third people’s hospital, Huzhou, China; Department of Plastic surgery, Second Affiliated Hospital, Zhejiang University School of Medicine, Hangzhou, China; Department of Oncology, Second Affiliated Hospital, Zhejiang University School of Medicine, Hangzhou, China

**Keywords:** Lgr5, Prognostic value, Clinical-pathological differences, Colorectal cancer, Meta-analysis

## Abstract

**Background:**

Lgr5 has recently been identified as a reliable biomarker of cancer stem cells (CSCs) in colorectal cancer (CRC); however, its prognostic value is still controversial.

**Methods:**

We searched PubMed, Web of Science, and Wanfang databases with identical strategies to retrieve articles. We evaluated the impact of Lgr5 expression on survival of CRC patients through meta-analysis.

**Results:**

A total of 12 studies comprising 2600 patients revealed that Lgr5 overexpression was negatively associated with overall survival (OS) (HR = 1.73, 95 % CI: 1.28–2.33; *P =* 0.00) and disease free survival (DFS) (HR = 2.89, 95 % CI: 1.89–4.44; *P =* 0.000) in CRC patients. Subgroup analysis suggested that Lgr5 overexpression was significantly associated with worse OS in subgroups with IHC as the method of Lgr5 assessment (HR = 2.01, 95 % CI: 1.39–2.89; *P =* 0.001), patients from Asia (HR = 1.81, 95 % CI: 1.27–2.58; *P =* 0.000), and NOS scores greater than 6 (HR = 2.12, 95 % CI: 1.41–3.19; *P =* 0.000). Furthermore, sensitivity analysis showed that the estimated HR ranged from 1.6 to 1.86 upon excluding one study sequentially from each analysis. In addition, Lgr5 overexpression was significantly associated with deep invasion of CRC (OR = 0.39, 95 % CI: 0.17–0.87; *P =* 0.002), lymphnode metastasis (OR = 0.45, 95 % CI: 0.26–0.76; *P =* 0.003), distant metastasis (OR = 0.37, 95 % CI: 0.22–0.62; *P =* 0.000), and AJCC stage (OR = 0.35, 95 % CI: 0.15–0.78; *P =* 0.01). However, Lgr5 overexpression was not correlated with tumor grade (OR = 0.75 95 % CI: 0.37–1.54; *P =* 0.433).

**Conclusions:**

This study shows that Lgr5 can be a valuable and reliable prognostic factor of colorectal cancer progression.

## Background

Colorectal cancer (CRC) is the third most common form of cancer and the third leading cause of cancer-related deaths in the United States [1]. Despite the enormous progress that has been made in CRC treatment, the overall mortality of CRC is still around 40 % [2]. Currently, TNM classification, including local tumor infiltration depth, lymph node involvement, and distant organ metastasis, is the main criterion for identifying those patients who carry a high risk for disease relapse and poor outcomes [3]. Unfortunately, 40–60 % of cases classified as either stage II or stage III will relapse or develop metastases following curative resection [4]. In addition, patients can follow significantly different clinical courses despite being diagnosed in the same stage. Therefore, identification of novel prognostic factors to distinguish high-risk groups is imperative for the improvement of therapeutic approaches in CRC treatment.

Recent mounting evidence indicates that cancer stem cells (CSCs) play a crucial role in tumor initiation, therapy resistance, disease relapse, and tumor progression [5–7]. A number of studies have demonstrated that expression of CSC markers in CRC have prognostic significance [8, 9]. CD133 was initially identified as a biomarker in primary CRC [10, 11] where it was believed to be widely expressed in human primary colon cancer epithelial cells. However, the CD133- subpopulation is now known to be composed mostly of stromal and inflammatory cells [12] that possess the ability to initiate xenograft tumors. Alternatively, Leucine-rich repeat-containing G-protein-coupled receptor 5 (Lgr5), a member of the G protein-coupled receptor (GPCR) family of proteins, has recently been reported as a reliable biomarker of CSCs in CRC [13]. Many studies have demonstrated that Lgr5 protein is overexpressed in CRC and is associated with tumor initiation, 5-FU-based chemotherapy resistance, and recurrence [14–18]. Therefore, Lgr5 expression is thought to be a potential biomarker related to poor prognosis in CRC. However, a recent, retrospective study comprising 891 colorectal adenocarcinomas revealed that Lgr5 did not have prognostic significance in CRC [19]. These contradictory results highlight the need to systematically analyze the data of Lgr5 expression in CRC to draw a reasonable conclusion about its prognostic significance.

In this study, we performed a meta-analysis to explore the association between Lgr5 expression and the prognosis of CRC. The correlation of Lgr5 expression with clinical-pathological features in CRC, such as tumor grade and tumor stage, was also investigated.

## Methods

### Literature search

We searched PubMed, Web of Science, and Wanfang databases using the following terms: “Lgr5”, “colorectal neoplasms”, “colorectal cancer”, “colon cancer”, “rectal cancer”, and “prognosis”. We also searched references cited in retrieved articles to identify additional eligible studies. The last search update was February 15^th^, 2014. In studies with overlapping patients, the most informative one was chosen to avoid duplicate information.

### Inclusion and exclusion criteria

The eligible studies included in our meta-analysis met the following inclusion criteria: (1) evaluated the relationship between Lgr5 expression in human CRC samples and overall survival (OS), disease free survival (DFS), or clinicopathological characteristics of CRC; (2) provided sufficient data to calculate hazard ratios (HRs) or odds ratios (ORs) and their 95 % confidence intervals (CIs); (3) published in English or Chinese. The following articles were excluded: (1) articles without original data such as letters, case reports, reviews, or conference abstracts; (2) articles published in a language other than English or Chinese; (3) articles that lacked the necessary data for estimating HRs or ORs and the corresponding 95 % CIs.

### Data extraction and assessment of study quality

Two authors independently reviewed and extracted data from each eligible study. Disagreements in data extraction were arbitrated by a third investigator. The following data were retrieved from the studies: name of the first author, year of publication, country of origin of the patients, number of patients included in the study, method used to detect Lgr5, cut-off value of Lgr5 expression, study design, clinicopathological features, and survival data. The quality of each eligible study was assessed according to the Newcastle–Ottawa quality assessment scale.

### Statistical analysis

To pool the survival data quantitatively, the impact of Lgr5 overexpression on OS or DFS of patients with CRC was evaluated by HRs with 95 % CIs. The HRs with 95 % CIs were estimated according to the methods reported by Parmar [20]. We used the raw data directly if HRs and their corresponding 95 % CIs were described in the publication. Otherwise, the value of HRs with 95 % CIs was estimated by other parameters, such as the O-E statistic and variance. Also, Kaplan-Meier curves of OS or DFS were read by Engauge Digitizer 4.1 (http://digitizer.sourceforge.net/). ORs (odds ratios) with 95 % CIs were used to determine the relationship between Lgr5 overexpression and clinicopathological parameters of CRC, such as tumor grade, AJCC stage, depth of invasion, lymph node metastasis, and distant metastasis. An observed HR > 1 and an observed OR < 1 indicated a worse prognosis for survival and unfavorable parameters in patients that overexpressed Lgr5. The impact of Lgr5 overexpression on survival or clinicopathological features was considered to be statistically significant if the 95 % CI did not overlap with 1.

The heterogeneity that exists in a meta-analysis due to the variation in outcomes between studies was evaluated by Chi-square, according to Peto’s method [21]. The inconsistency index (I^2^) statistic (range from 0 % to 100 %) was used to quantify the percentage of total variation across studies that is due to heterogeneity rather than sampling error [22]. A *P <* 0.10 for the Q-test indicated heterogeneity exists among the studies, then we used the random-effects model (the DerSimonian and Laird method) [22]. Otherwise, we adopted the fixed-effects model (Mantel-Haenszel) to calculate the pooled ORs and HRs [22]. Both the Begg’s test and Egger’s test were used to determine publication bias where *P <* 0.05 indicated bias. All calculations were performed using STATA 12.0 software (Stata Corporation, Collage Station, Texas, USA). A *p*-value < 0.05 was considered statistically significant.

## Results

### Search results and description of the studies

**Fig. 1:**
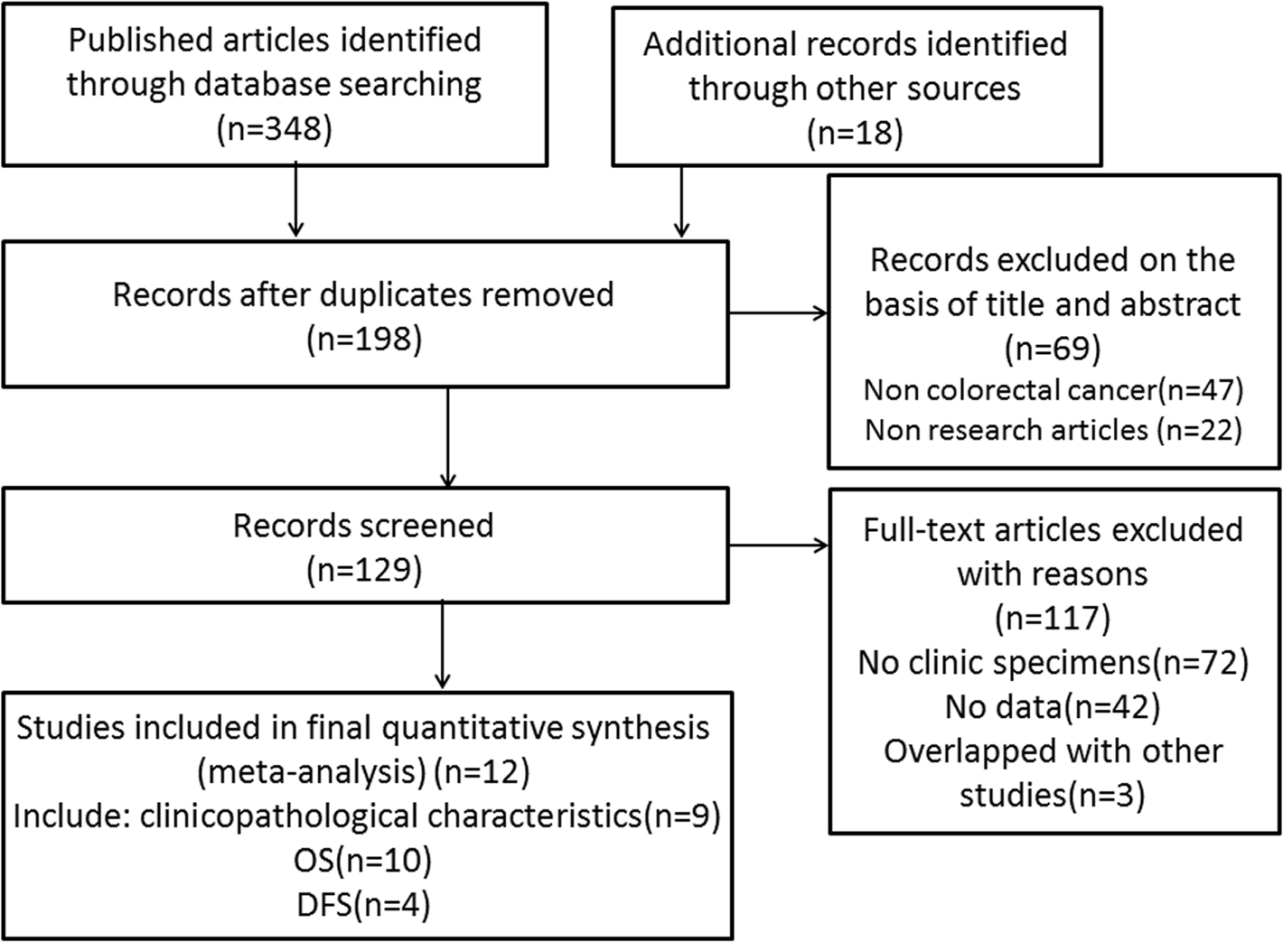
Flow diagram of the selection procedure for the studies

Upon analysis of the title and abstract of 348 potential studies that were retrieved using the search strategy described above, we identified 129 articles involving the survival risk of CRC and Lgr5 expression. Of these 129 studies, 42 did not offer sufficient data to calculate the HR or OR, three overlapped with other studies, and 72 did not involve clinical specimens resulting in a total of twelve studies that were included in our meta-analysis [14, 19, 23–32]. In addition, no another study was identified through searching references cited in retrieved articles. A detailed search strategy is described in Fig. 1.

The characteristics of the nine eligible studies, which were retrospective case–control studies published between 2010 and 2014, are summarized in Table 1. This meta-analysis involved a total of 2600 CRC patients with sample sizes ranging from 44 to 891 patients. Of the twelve studies, seven evaluated patients from China, three assessed patients from Japan,and one each examined patients from America and Spain. Furthermore, four studies assessed the relationship between Lgr5 expression and DFS of CRC patients while ten studies evaluated the correlation between Lgr5 expression and OS of CRC patients. With regards to the method of analysis, nine studies assessed Lgr5 expression using immunohistochemistry (IHC), two determined Lgr5 expression by RT-PCR, and the remaining study evaluated Lgr5 expression through in situ hybridization (ISH).

**Table 1:** Characteristics of the studies included for the meta-analysis

First author	Year	Country	Patient (M/F)	Age (year)	Technology	Lgr5 positive threshold	HR (95 % CI) of OS	HR (95 % CI) of DFS	Quality score
Liu	2014	China	363(200/166)	59mean	IHC	NA	2.604 (1.894–3.584)	NA	7
He	2014	China	53(29/24)	NA	IHC	NA	2.482 (1.52–4.283)	NA	7
Gao	2014	China	44(22/20)	59median	IHC	The percentage of stained cells ≥ 10 %	2.299 (0.922–5.714)	NA	6
Wu	2012	China	192(120/72)	62median	IHC	Multiplying the intensity and the quantity score > 5	2.768 (1.619–4.732)	NA	8
Hsu	2013	China	296(169/127)	63.5mean	IHC	The percentage of stained cells multiplied by the intensity scores ≥180	NA	2.21 (1.11–4.37)	7
Saiqusa	2012	Japan	52(41/11)	64.5median	IHC	The percentage of stained cells ≥ 50 %	1.061 (0.299–3.771)	4.942 (1.39–17.577)	6
Ziskin	2012	America	891(467/424)	71.4mean	ISH	Intensity score >1	1.15 (0.95–1.4)	NA	7
Valladares-Ayerbes	2012	Spain	54(33/21)	62.7mean	QRT-PCR	11.6	2.517 (0.924–6.858)	2.995 (1.192–7.527)	8
Takahashi	2011	Japan	180(105/75)	NA	QRT-PCR	Median value	0.909 (0.535–1.543)	3.3 (1.49–7.33)	6
Bao	2012	China	246(131/115)	63mean	IHC	The percentage of stained cells ≥ 5 %	1.122 (0.866–1.448)	NA	4
Peng	2010	China	169(101/68)	57mean	IHC	The percentage of stained cells ≥ 10 %	2.16 (1.35–3.45)	NA	7
Takeda	2010	Japan	60(32/28)	65.7mean	IHC	The percentage of stained cells ≥ 5 %	NA	NA	7

### Methodological quality of the studies

We assessed the quality of the 12 eligible case–control studies according to the Newcastle–Ottawa Scale (NOS), a method developed as a collaborative effort between the Universities of Newcastle, Australia and Ottawa, Canada. NOS scores were determined by judging the studies on items in three general categories, including the selection of the study populations, the comparability of the populations, and the ascertainment of either the exposure or the outcome of interest. Scores ranged from 0 (lowest) to 9 (highest), and studies with scores of 7 or more were defined as high quality studies. The quality scores of each study are summarized in Table 1. The median and mean score of these 12 studies were 7 and 6.67, respectively, indicating that they were of high quality.

### Correlation of Lgr5 overexpression with decreased OS and DFS in CRC

**Fig. 2:**
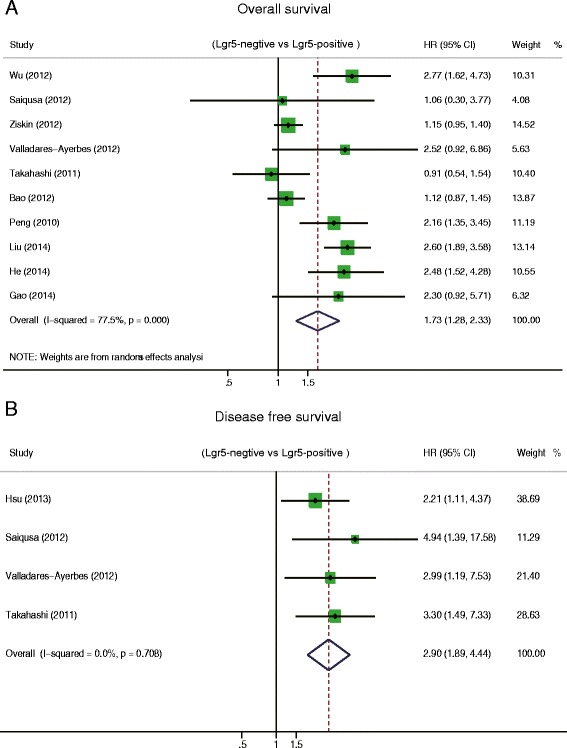
Hazard ratio (HR) of Lgr5 overexpression associated with (**a**) overall survival (OS) and (**b**) disease free survival (DFS)

We performed a meta-analysis on ten studies to evaluate the association between Lgr5 overexpression and OS and a meta-analysis on four studies to determine the association between Lgr5 overexpression and DFS. The pooled hazard ratio (HR) for OS was 1.73 (95 % CI: 1.28–2.33; Z = 3.59; *P =* 0.00) (Fig. 2) with heterogeneity (I^2^ 77.5 % *P =* 0.00), indicating that the HR of overall death was 1.73-fold higher in patients with increased levels of Lgr5; the pooled HR for DFS was 2.89 (95 % CI: 1.89–4.44; Z = 4.89; *P =* 0.000) without heterogeneity (I^2^ 0 % *P =* 0.708) (Fig. 2). These results suggest that Lgr5 overexpression is significantly correlated with a worse prognosis in CRC patients.

### Subgroup analysis and sensitivity analysis of the relationship between Lgr5 overexpression and OS in CRC

**Fig. 3:**
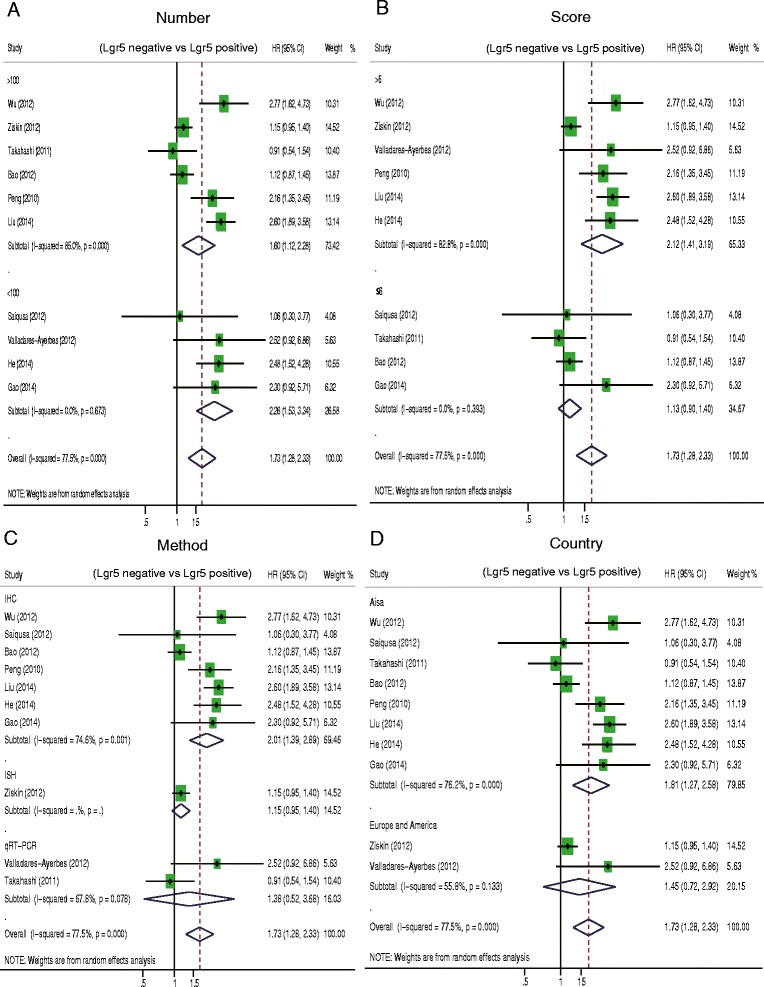
Hazard ratio (HR) of Lgr5 overexpression associated with overall survival (OS) in the subgroup of (**a**) patient sample size, (**b**) NOS score of study, (**c**) the Lgr5 assessment method, and (**d**) patients’ country of origin

We performed subgroup analysis and sensitivity analysis in order to address the heterogeneity that was observed in the correlation between Lgr5 overexpression and decreased OS in CRC patients. The characteristics that we evaluated for the subgroup analysis were the following: number of patients involved in the study, the country of origin of the patients, the method used to determine Lgr5 expression, and NOS scores of the study. Lgr5 overexpression was correlated with worse OS in subgroups with patients from Asia (HR = 1.81, 95 % CI: 1.27–2.58; *P =* 0.000), IHC as a method of Lgr5 assessment (HR = 2.01, 95 % CI: 1.39–2.89; *P =* 0.001), and NOS scores greater than 6 (HR = 2.12, 95 % CI: 1.41–3.19; *P =* 0.000) (Fig. 3) (Table 2). We also conducted a sensitivity analysis to evaluate the effect of a single study on the overall estimate by sequentially excluding each study, as outlined in Table 2. Upon omitting each of the indicated studies, the estimated HR ranged from 1.6 to 1.86 (Table 3).

**Table 2:** Subgroup analysis of pooled hazard ratios of colorectal patients with Lgr5 overexpression

					Heterogeneity		
Stratified analysis	Number of studies	Number of patients	Pooled HR (95 % CI)	*P* value	I^2^(%)	*P* value	Interaction *p* value
Study location							0.518
Asia	8	1299	1.81 (1.27–2.58)	0.001	76.2	0.000	
Europe and America	2	945	1.45 (0.72–2.92)	0.299	55.8	0.133	
Number of patients							0.38
>100	6	2041	1.6 (1.12–2.29)	0.01	85	0.043	
<100	4	203	2.26 (1.53–3.34)	0.000	0	0.673	
Method of measurement							0.15
IHC	7	1119	2.01 (1.39–2.89)	0.000	74.6	0.001	
ISH	1	891	1.15 (0.95–1.4)	0.158			
qRT-PCR	2	234	1.38 (0.52–3.68)	0.521	67.8	0.078	
NOS score							0.055
>6	6	1669	2.12 (1.41–3.19)	0.000	82.8	0.000	
≦6	4	575	1.13 (0.9–1.4)	0.291	55.9	0.393

**Table 3:** Meta sensitivity analysis of Lgr5 expression and OS

Study omitted	Estimated HR	Low value of 95 % CI	High value of 95 % CI
Wu (2012) [23]	1.6325674	1.2023937	2.2166421
Saiqusa (2012) [24]	1.7648035	1.295853	2.4034605
Ziskin (2012)	1.8513726	1.3232487	2.5902767
Valladares-Ayerbes (2012) [25]	1.6885946	1.2394606	2.300478
Takahashi (2011) [26]	1.8601217	1.3568895	2.5499887
Bao (2012) [29]	1.8537774	1.3164659	2.6103909
Peng (2010) [28]	1.6803607	1.2180028	2.3182311
Liu (2014) [32]	1.6021169	1.2043179	2.1313131
He (2014) [31]	1.6534556	1.2091391	2.2610431
Gao (2014) [30]	1.6943128	1.2407216	2.3137312
Combined	1.7265576	1.2812517	2.3266318

### Impact of Lgr5 overexpression on clinicopathological features of CRC

**Fig. 4:**
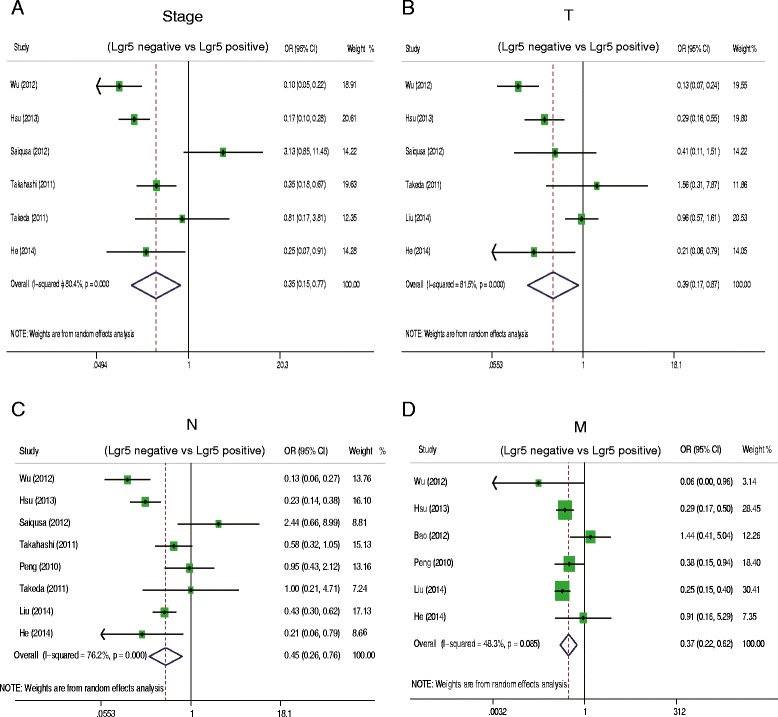
Odds ratio (OR) of Lgr5 overexpression associated with clinicopathological features of colorectal cancer. **a** The pooled OR and its corresponding 95 % CI of Lgr5 overexpression with AJCC stage. **b** The pooled OR and its corresponding 95 % CI of Lgr5 overexpression with primary tumor. **c** The pooled OR and its corresponding 95 % CI of Lgr5 overexpression with lymph node  metastasis. **d** The pooled OR and its corresponding 95 % CI of Lgr5 overexpression with distant metastasis

Next, we examined the relationship between Lgr5 overexpression and several clinicopathological parameters of CRC (Fig. 4). Lgr5 overexpression appeared to be significantly associated with deep invasion of CRC (OR = 0.39, 95 % CI: 0.17–0.87; *P =* 0.002), lymph node metastasis (OR = 0.45, 95 % CI: 0.26–0.76; *P =* 0.003), distant metastasis (OR = 0.37, 95 % CI: 0.22–0.62; *P =* 0.000), and AJCC stage (OR = 0.35, 95 % CI: 0.15–0.78; *P =* 0.01). (Fig. 4); however, it was not correlated with tumor grade (OR = 0.75 95 % CI: 0.37–1.54; *P =* 0.433).

### Publication bias

**Fig. 5:**
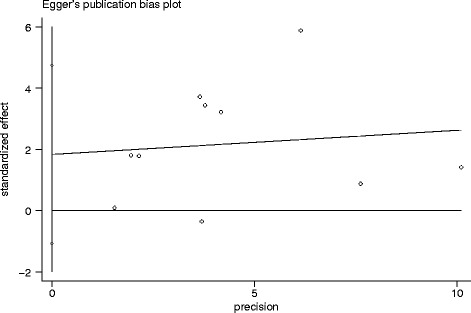
Egger’s publication bias plot. It showed no publication bias for studies regarding the association of Lgr5 with overall survival (OS) in the meta-analysis. Each point represents a separate study for the indicated association

We assessed the potential publication bias both graphically, through funnel plots of the Egger’s test on OS (Fig. 5), and statistically, by the Egger’s and Begg’s test. The symmetry of the funnel plots and the *p*-values from the statistical analysis suggest no publication bias.

## Discussion

Lgr5, also known as GPR49, has been reported to be a reliable biomarker of CSCs in CRC [13]. Overexpression of Lgr5, through an in vitro assay, resulted in enhanced proliferation and resistance to chemotherapy [14, 18]. Consistent with these results, Lgr5 ablation in CRC cell lines inhibited cell growth, enhanced apoptosis, and increased the sensitivity of cells to chemotherapy [14]. The close relationship between Lgr5 and Ki-67 further supports the correlation between Lgr5 overexpression and increased proliferative capability [23]. Furthermore, Lgr5 was recently shown to be involved in the carcinogenesis of CRC as a target of the Wnt signaling pathway [33, 34]. Despite these data, the relationship between Lgr5 expression and prognosis of CRC is still not completely understood and Lgr5, as a useful biomarker associated with poor prognosis in CRC, still remains controversial. Therefore, in this study, we performed a meta-analysis to systematically evaluate the association between Lgr5 expression and the prognosis of CRC.

Previous studies reported that Lgr5 was overexpressed in human colon tumors, as compared to normal colon tissues [16]. Lgr5 expression was also significantly correlated with worse prognosis in 192 CRC patients by immunohistochemistry [23]. Consistent with these findings, our meta-analysis demonstrated that elevated Lgr5 expression was negatively associated with OS and DFS in CRC patients, suggesting an important role for Lgr5 in tumor progression. Subgroup analysis further distinguished that Lgr5 overexpression was correlated with worse OS when patients from Asia,IHC as a method of Lgr5 assessment and NOS scores were greater than 6. The outcome of the sensitivity analysis corroborated the relationship between Lgr5 overexpression and worse OS in CRC patients. Furthermore, several studies showed that the expression of Lgr5 was up-regulated in advanced CRC [14, 17, 26]. The relationship between Lgr5 expression and clinicopathological parameters was analyzed, and the results showed that Lgr5 overexpression was significantly correlated with deep invasion, lymph node metastasis,distant metastasis, and advanced AJCC stage. These results suggest that Lgr5 expression could serve as a valuable prognostic factor for CRC patients.

However, inevitably, some limitations exist in our meta-analysis. First, although random effects model was used to deal with heterogeneity, the inter-study heterogeneity caused by the use of different populations, varying detection methods, and different cutoff values was inevitable. Second, the number of studies included in subgroup analysis according to country and measurement method was relatively small, it may result in bias and further studies will need to be performed in order to confirm and strengthen these results. Finally, the results of retrospective case–control studies in our meta-analysis were less reliable than that of prospective cohort studies or randomized controlled trials.

## Conclusion

In conclusion, our results show that Lgr5 overexpression is significantly associated with poor OS as well as DFS of CRC patients. Furthermore, advanced AJCC stage, deep invasion, and distant metastasis seems to be more frequent in patients that overexpress Lgr5. Therefore, Lgr5 overexpression appears to be a valuable prognostic factor and a reliable indicator of CRC progression.
